# Unexplained death in patients with *NGLY1* mutations may be explained by adrenal insufficiency

**DOI:** 10.14814/phy2.13979

**Published:** 2019-02-10

**Authors:** Britt J. van Keulen, Joost Rotteveel, Martijn J. J. Finken

**Affiliations:** ^1^ Emma Children's Hospital Amsterdam UMC Vrije Universiteit Amsterdam Pediatric Endocrinology Amsterdam The Netherlands

**Keywords:** Adrenal insufficiency, NGLY1 mutation, proteotoxic stress

## Abstract

Homozygous mutations in *NGLY1* were recently found to cause a condition characterized by a complex neurological syndrome, hypo‐ or alacrimia, and elevated liver transaminases. For yet unknown reasons, mortality is increased in patients with this condition. *NGLY1* encodes the cytosolic enzyme N‐glycanase 1, which is responsible for the deglycosylation of misfolded N‐glycosylated proteins. Disruption of this process is hypothesized to lead to an accumulation of misfolded proteins in the cytosol. Here, we describe the disease course of a girl with a homozygous mutation in *NGLY1*, namely c.1837del (p.Gln613 fs). In addition to the previously described symptoms, at the age of 8 she presented with recurrent infections and hyperpigmentation, and, subsequently, a diagnosis of primary adrenal insufficiency was made. There are no previous reports describing adrenal insufficiency in such patients. We postulate that patients with NGLY1 deficiency may develop adrenal insufficiency as a consequence of impaired proteostasis, and the accompanying proteotoxic stress‐induced cell death, through defective Nrf1 function. We recommend an annual evaluation of adrenal function in all patients with *NGLY1* mutations in order to prevent unnecessary deaths.

## Abstract 2

Homozygous mutations in NGLY1 were recently found to cause a condition characterized by a complex neurological syndrome, hypo‐ or alacrimia, and elevated liver transaminases. For yet unknown reasons, mortality is increased in patients with this condition. We postulate that patients with NGLY1 mutations may develop adrenal insufficiency as a result of proteotoxic stress‐induced cell death, which could be an explanation for this increased mortality.

## Introduction

A causal link between loss‐of‐function mutations in *NGLY1* and a complex neurological syndrome was first described in 2012 (Need et al. 2012). *NGLY1* encodes the cytoplasmic peptide N‐glycanase 1, an enzyme responsible for the deglycosylation of misfolded N‐glycosylated proteins, which are retrotranslocated from the endoplasmic reticulum to the cytoplasm (Suzuki et al. 1993; Hirsch et al. 2003; Suzuki 2015). Disruption of this process might result in an abnormal accumulation of misfolded glycoproteins. The clinical phenotype of *NGLY1* mutations includes a complex neurological syndrome (Enns et al. 2014). Persons with *NGLY1‐*related disorders typically show developmental delay, hypotonia, a hyperkinetic movement disorder, hypo‐ or alacrima, and elevated liver transaminases. The latter is typically observed at a young age but does not necessarily persist. Approximately, half of the patients have clinical seizures. In addition, sleep apnea, oral motor defects that affect feeding ability, auditory neuropathy, constipation, scoliosis, and peripheral neuropathy have been described (Lam et al. 2018). For yet unknown reasons, mortality is increased in patients with this condition. Until now, the combination of an *NGLY1* mutation and impaired adrenal function has not been described in literature. In this report, we describe a patient with a homozygous mutation in *NGLY1*, namely c.1837del (p.Gln613fs), who presented with adrenal insufficiency at the age of 8 years.

## Clinical Report

Our patient was born after a pregnancy duration of 38 weeks with a birth weight of 2100 g (<−2 SD score). She was the second child of consanguineous parents (full cousins). During the first months of life, her psychomotor development was virtually normal. From the age of 3 months, her development started to lag behind. Her clinical symptoms were no different from those of other patients with *NGLY1* mutations, and included severe psychomotor retardation, seizures, scoliosis, and oral motor defects. Whole exome sequencing was conducted, which demonstrated a homozygous mutation in *NGLY1*, namely NGLY1_NM_001145293.1:c.1837del p.(Gln613fs) (chr3.hg19:g25761025del).

At the age of 8 years, our patient experienced several severe respiratory infections, for which she was admitted to the intensive care unit several times. In addition, the parents reported increased vomiting and a bronze tint. On physical examination, hyperpigmentation of especially the skin folds was noticed. Due to clinical suspicion of adrenal insufficiency, laboratory analysis was conducted, demonstrating a cortisol level below the detection limit in conjunction with adrenocorticotropic hormone (ACTH) excess, suggestive of primary adrenal insufficiency. A standard ACTH1‐24 stimulation test showed no rise of cortisol, confirmative of adrenal insufficiency. Renin concentration was elevated, indicative of aldosterone deficiency (Table 1). The adrenals were not visible on ultrasound investigation, suggesting loss of adrenal tissue. After the initiation of hydrocortisone and fludrocortisone replacement therapy, her clinical symptoms improved.

**Table 1:** Laboratory results of our patient

Parameter	Value	Reference range
ACTH	515	<9 pmol/L
Cortisol	<13.8	150–600 nmol/L
Cortisol t0 min^1^	<30	150–600 nmol/L
Cortisol t30 min^1^	<30	150–600 nmol/L
Cortisol t60 min^1^	<30	150–600 nmol/L
Aldosterone	0.33	0.03–0.54 nmol/L
Renin	176	5–45 mU/L

1 During an adrenocorticotropic hormone stimulation test.

## Discussion

We have described a girl with a homozygous *NGLY1* mutation and proven adrenal insufficiency. To the best of our knowledge, the combination of *NGLY1* mutation and adrenal insufficiency has never been described. In current clinical practice, adrenal function is not evaluated in patients with *NGLY1* mutations. It is possible that the increased mortality risk associated with *NLGY1* mutation is explained by undiagnosed adrenal insufficiency.

We propose a causal link between *NGLY1* mutation and adrenal insufficiency. Several patients with an *NGLY1* mutation had died unexpectedly during infancy. One of them deceased after a viral infection complicated by a prolonged seizure at the age of 5 years (Enns et al. 2014). Another child died unexpectedly in her sleep at the age of 9.5 months and the cause of death has remained unknown (Enns et al. 2014). A third child suffered from recurrent respiratory infections and deceased from respiratory failure at the age of 16 years (Caglayan et al. 2015). Two of the deceased children were found to have significant adrenal cortex vacuolization and low unconjugated estriol (uE3) (Enns et al. 2014). Unfortunately, adrenal function was never evaluated in these patients. It is likely that these patients had died from undiagnosed adrenal insufficiency.

The pathophysiologic mechanism behind the adrenal insufficiency is not yet elucidated. Irreparably misfolded proteins are tagged for degradation via endoplasmic reticulum‐associated degradation. This is an essential quality control system for glycoproteins in the endoplasmic reticulum. N‐glycanase 1 is responsible for the deglycosylation of misfolded proteins in the endoplasmic reticulum by cleavage of the aspartyl glycosylamine bond of *N‐*linked glycoproteins. Disruption of this process might result in an accumulation of intact glycoproteins that cannot undergo further processing in the cytoplasm. Trapping of glycoproteins in cells may manifest itself as vacuoles, as was seen in the adrenal cortex of deceased patients with *NGLY1* mutations (Enns et al. 2014). In addition, liver tissue obtained by biopsy in a patient with an *NGLY1* mutation showed an amorphous unidentified substance throughout the cytoplasm, suggestive of accumulated material (Need et al. 2012). These organs may be particularly vulnerable for the accumulation of glycoproteins, given their function in protein synthesis.

Recent studies demonstrated the importance of NGLY1 for the regulation of proteostasis and mitochondrial homeostasis (Tomlin et al. 2017; Yang et al. 2018). NGLY1 is essential for the activation of Nuclear Factor Erythroid 2 like 1, also referred to as Nrf1 (Tomlin et al. 2017). Nrf1, in turn, has been implicated to play a crucial role in a host of cellular functions, including oxidative stress response, differentiation, inflammatory response, and metabolism, in addition to maintenance of proteostasis (Kim et al. 2016). When proteasome capacity is saturated, Nrf1 accumulates in the cytosol. Here, it is activated through de‐N‐glycosylation and proteolytic processing by N‐glycanase 1 and DDI2, respectively. Activated Nrf1 migrates to the nucleus, where it mediates a “bounce‐back” response by upregulating proteasome subunit gene expression (Radhakrishnan et al. 2010; Lehrbach and Ruvkun 2016; Owings et al. 2018). In NGLY1 deficiency, Nrf1 is inactive in regulating proteasome subunit gene expression in response to proteasome insufficiency. This was corroborated by findings from studies in various leukemia cell lineages, demonstrating that chemical inhibition of NGLY1‐potentiated cytotoxicity caused by proteasome inhibition (Tomlin et al. 2017). In normal circumstances, NGLY1 is highly expressed in adrenal cells, especially those in the cortex, which might imply that it fulfills a crucial function here (Lindskog 2015). The proteotoxic stress‐induced loss of adrenal cortex cells is thought to ultimately result in glucocorticoid and mineralocorticoid deficiency.

In our patient, who initially presented with developmental delay, a genetic diagnosis was established prior to the onset of the adrenal insufficiency. In patients with a preexistent complex neurological syndrome of yet unknown cause and a new diagnosis of adrenal insufficiency, several diagnoses other than NLGY1 deficiency should be considered as well, as described previously (Lam et al. 2018). Notably, the phenotypic spectrum of Triple‐A syndrome (or Allgrove syndrome), caused by homo‐ or compound heterozygous mutations in *AAAS* (encoding Aladin), shows a striking resemblance with that of NLGY1 deficiency. The majority of patients with Triple‐A syndrome suffer from progressive neurodegenerative symptoms, in addition to the 3 A's: adrenal insufficiency, alacrima, and achalasia. Adrenal failure associated with Triple‐A syndrome typically becomes manifest in the first decade as isolated glucocorticoid deficiency (Roucher‐Boulez et al. 2018). Some mitochondrial diseases, for example, IARS2‐related mitochondrial disease, may also manifest adrenal insufficiency (Vona et al. 2018). However, the combination of mitochondrial disease and adrenal insufficiency has only rarely been described (Schaefer et al. 2013).

## Conclusion

This case report, along with previous reports on significant vacuolization of adrenal cortex in other NGLY1 deficiency patients, suggests that a causal link might exist between *NGLY1* mutation and adrenal insufficiency. More research is needed to elucidate the exact pathophysiologic mechanism. At this point, we cannot exclude that the adrenal insufficiency observed in our patient was an incidental event not related to NGLY1 deficiency. However, since this is a potentially life‐threatening complication that can be readily addressed by hormone replacement therapy, we recommend evaluation of adrenal function in all patients with an *NGLY1* mutation. In our opinion, adrenal function should be evaluated annually or earlier when adrenal insufficiency is suspected on clinical ground.

## Conflict of Interest

None declared.
